# The role of meaningful work and workplace attachment styles in predicting entrepreneurial well-being: insights from a structured sales network

**DOI:** 10.3389/fpsyg.2025.1554683

**Published:** 2025-05-16

**Authors:** Vladimír Koša, Elena Lisá

**Affiliations:** Faculty of Social and Economic Sciences, Comenius University Bratislava, Bratislava, Slovakia

**Keywords:** meaningful work, workplace attachment, entrepreneurial well-being, structured sales network, leadership, financial performance, psychological safety

## Abstract

**Introduction:**

The study explores how meaningful work (MW) and workplace attachment styles, secure (SE), anxious (AX), and avoidant (AV), predict entrepreneurial well-being within a structured sales network of a financial services firm. It further examines the moderating role of attachment styles and the contextual influence of regional leadership and team financial performance.

**Methods:**

Data were collected from 300 entrepreneurs using the Work and Meaning Inventory (WAMI), the Workplace Attachment Questionnaire (WAQ), and the Well-Being Index (WHO-5).

**Results:**

Regression analyses show that MW is a robust and consistent predictor of well-being, even after controlling for team-level sales performance and gender. AX initially appeared to amplify the positive relationship between MW and well-being, but this interaction effect diminished once objective financial performance metrics were included. SE to leaders was positively associated with well-being in psychological models but lost significance in the extended model, suggesting that financial performance may partially mediate its effects. Well-being varied significantly by regional leadership, even after adjusting for sales outcomes.

**Discussion:**

The findings underscore the importance of promoting MW, monitoring leadership impact across regions, and integrating subjective and objective indicators when assessing entrepreneurial well-being.

## Introduction

Meaningful work (MW), often defined as work perceived to be inherently valuable, purposeful, and significant, has garnered substantial academic attention due to its positive impact on personal and organizational outcomes (Steger et al., 2012; Steger and Dik, 2009). Research, including meta-analytic evidence, indicates that individuals who perceive their work as more meaningful report higher levels of well-being and job satisfaction (Allan et al., 2019; Martela and Pessi, 2018). Additionally, they experience reduced levels of depression (Allan et al., 2016) and enhanced work engagement (Steger et al., 2013). Meaningfulness is thus regarded as a crucial psychological resource that fosters resilience and mitigates burnout risk (Allan et al., 2014).

Given these documented benefits, it is important to understand how MW is experienced across different occupational contexts. One context of increasing interest is entrepreneurship. Entrepreneurs are often perceived as experiencing higher levels of MW than organizational employees (Schwarz and Wahl, 2023). Entrepreneurs encounter unique stressors, such as financial uncertainty, fluctuating workloads, and the need for rapid adaptation to dynamic markets, which heighten the potential importance of MW in sustaining well-being. Freelancers and independent contractors, for instance, often enjoy significant autonomy, enabling them to align work activities with personal values, which may enhance their sense of MW (Rosso et al., 2010).

However, the entrepreneurial landscape is far from uniform. Not all entrepreneurs operate independently; many work within structured systems, such as distribution networks or franchise models tied to a single firm. In such settings, they navigate hierarchical relationships with managers and regional directors, which may constrain autonomy and shape their experiences of MW in more complex ways (Geldenhuys and Johnson, 2021).

The hierarchical entrepreneurial structure highlights the need to consider individual, contextual, and interpersonal factors. Among these, leadership quality emerges as particularly influential. Leadership can shape meaning-making by providing emotional support, articulating purpose, and modeling values. For example, transformational leadership has been associated with higher levels of MW and psychological safety, particularly among individuals with insecure attachment styles (Jiang et al., 2019). Thus, leadership is a critical contextual variable for understanding how MW translates into well-being in entrepreneurial settings.

For entrepreneurs, MW is often constructed through interpersonal cues that signal value, care, and security (Lysova et al., 2022). Supportive and high-quality relationships are positively associated with MW, which fosters an affective commitment to the organization (Hall et al., 2023). In startup environments, the workgroup and leaders can act as mentors or sources of inspiration, cultivating an atmosphere that fosters MW. These social dynamics influence how individuals perceive the value and meaning of their daily work activities (Vitória et al., 2024).

### Meaningful work and well-being

Extensive research highlights that MW is closely tied to psychological well-being, job satisfaction, and overall life fulfillment (Steger and Dik, 2009). The Work and Meaning Inventory (WAMI; Steger et al., 2012) measures MW through three core dimensions: positive meaning in work, creating meaning through one’s efforts, and the aspiration to contribute to the greater good. Meta-analyses underscore the predictive role of MW in numerous positive outcomes, including reduced depressive symptoms, enhanced engagement, and job satisfaction (Allan et al., 2019; Martela and Pessi, 2018).

Longitudinal studies demonstrate that higher MW levels strongly predict improved mental health over time, particularly among women and white-collar professionals (Herr et al., 2023). Additionally, MW plays a vital role in the well-being of professionals in specialized fields. For example, veterinarians find MW in activities that promote self-actualization, assist animals and humans, and foster community belonging. These factors align with the principles of eudaimonic well-being, emphasizing the crucial role of meaningfulness in supporting mental health and professional satisfaction (Wallace, 2019).

MW is a critical resilience buffer, mitigating stress and depressive symptoms while promoting overall mental health and adaptive coping (Allan et al., 2016). MW as a resilience buffer is particularly relevant in entrepreneurial contexts, where individuals routinely face uncertainty, heavy workloads, and emotional distress. For entrepreneurs, perceiving their work as meaningful can enhance motivation, increase engagement, and maintain psychological well-being in the face of such demands (Allan et al., 2018; Steger et al., 2013).

From an organizational perspective, MW has also been associated with improved job performance, increased intrinsic motivation, and stronger affective commitment to the organization. However, it is important to recognize that the pursuit of MW is not universally positive. In contexts where fair working conditions are absent, pursuing meaningfulness can paradoxically contribute to overwork, self-exploitation, and adverse health outcomes (Soren and Ryff, 2023). Thus, the promotion of MW must be accompanied by attention to structural and ethical working conditions.

### Meaningful work in entrepreneurial contexts

Recent research highlights that founders and entrepreneurial teams often experience higher levels of MW than employees in traditional organizational settings. The distinction largely stems from entrepreneurs’ ability to shape their work environments and align these with their values and goals (Schwarz and Wahl, 2023). Entrepreneurs, particularly freelancers and independent contractors, derive a substantial portion of their MW from a clear sense of purpose (Geldenhuys and Johnson, 2021). Entrepreneurs’ autonomy in decision-making significantly facilitates aligning work activities with personal aspirations, enhancing the overall sense of meaningfulness (Rosso et al., 2010). Moreover, their capacity to create or modify roles within their ventures further strengthens their sense of purpose and fulfillment (Geldenhuys et al., 2014). The alignment between personal values and work activities becomes particularly significant for entrepreneurs operating within structured organizational systems. While these systems may impose hierarchical constraints, they still allow for a degree of self-governance that enables entrepreneurs to shape their roles meaningfully.

Employees in traditional organizations often face limitations in reshaping their roles, restricting their ability to experience MW compared to founders and entrepreneurial teams. Entrepreneurial teams, especially co-founders, report notably higher levels of MW than those in other organizational structures. Their heightened sense of meaningfulness is attributed to their active involvement in designing and influencing their work environments (Schwarz and Wahl, 2023). Conversely, employees in traditional settings often struggle with organizational constraints that hinder role customization and creative expression.

For self-employed individuals, the purpose remains a cornerstone of MW. Their ability to express creativity and engage in fulfilling activities underscores the critical role of autonomy in fostering meaningfulness (Geldenhuys and Johnson, 2021). Autonomy enhances satisfaction and facilitates sustained engagement in the work, further contributing to the richness of their professional experiences.

### Meaningful work and workplace attachment styles

Attachment theory posits that individuals develop emotional bonds in workplace settings analogous to those in personal relationships (Hazan and Shaver, 1990; Harms, 2011). Secure attachment (SE) fosters an environment of trust, emotional safety, and openness. Conversely, anxious attachment (AX) is characterized by a fear of rejection and heightened sensitivity to interpersonal cues. In contrast, avoidant attachment (AV) is marked by discomfort with closeness and a preference for self-reliance.

In workplace contexts, individuals with AX may seek reassurance and validation. They often find positive cues, such as perceiving their work as meaningful, particularly salient (Mikulincer and Shaver, 2007). MW can mitigate the adverse effects of AX, enabling employees to respond more favorably despite attachment insecurities (Jiang et al., 2019). While direct studies linking MW to workplace attachment styles are limited, insights from research on life meaning provide valuable perspectives. Securely attached individuals report a stronger presence of meaning in life compared to other attachment styles, while dismissive individuals demonstrate higher life meaning than their preoccupied or fearful counterparts (Bodner et al., 2014). Fearful and preoccupied individuals, however, tend to prioritize the search for meaning, often as a compensatory mechanism to address their insecurity. These patterns suggest that AX may lead individuals to emphasize finding meaning in life and work contexts more than securely or avoidantly attached individuals.

Jiang et al. (2019) found that MW moderates the relationship between insecure attachment styles (anxious and avoidant) and work outcomes. Their mediation analysis revealed that MW enhances the positive effects of transactional leadership, enabling employees with insecure attachment styles to respond more effectively to leadership. Specifically, perceiving work as meaningful improved employees’ psychological well-being and job satisfaction.

As defined by Edmondson (1999), psychological safety amplifies the positive effects of MW in the workplace (Rabiul et al., 2024). Psychological safety predicts MW and mediates the relationship between transformational leadership and MW. However, negative interpersonal dynamics, such as incivility or customer hostility, can undermine psychological safety, weakening its connection to MW. These findings underscore the importance of fostering a psychologically safe environment while minimizing adverse interpersonal interactions to support MW experiences.

Workplace attachment styles often align with affective commitment, which refers to the emotional bonds employees form with their organizations (Meyer and Allen, 1991). MW positively correlates with affective commitment. For example, in industries such as port services, employees who perceive their work as more meaningful report higher organizational commitment and job satisfaction (Firdausi and Prabandini Mulyana, 2024). Employees with SE styles are particularly likely to find their work meaningful due to their positive attitudes, self-efficacy, trust in leadership, and strong organizational commitment (Pham et al., 2023; Warnock et al., 2024).

Given these theoretical underpinnings, it is important to examine how MW and workplace attachment styles jointly relate to entrepreneurial well-being, especially in hybrid work settings where autonomy coexists with hierarchical structures. The current study addresses this gap by examining entrepreneurs working as freelancers within a structured sales network of a large international financial services firm. Despite their independent status, these individuals function within a hierarchical framework, reporting to managers (also independent) and regional directors (corporate employees). This mixed entrepreneurial independence and organizational constraint environment provides a unique context for examining how MW, well-being, and attachment styles interact.

The current study also considers leadership, specifically the role of regional leaders within the structured sales network, as a contextual variable that may influence entrepreneurial well-being. While not direct supervisors, these leaders serve as points of contact, guidance, and symbolic authority. Their behavior can either facilitate or impede the experience of MW, especially for individuals with different workplace attachment styles. Therefore, leadership is examined as a source of contextual variability and psychological influence within the entrepreneurial environment. Given their central position within regional networks, regional leaders may significantly influence how MW is perceived and how it translates into well-being outcomes.

Specifically, the study examines whether attachment styles predict or moderate the relationship between MW and well-being. Based on previous findings that MW can mitigate the adverse effects of insecure attachment (Jiang et al., 2019), the study formulates the following hypotheses:

*H1*: Meaningful work (MW) positively predicts entrepreneurial well-being (Allan et al., 2019; Martela and Pessi, 2018; Allan et al., 2018; Steger et al., 2013).

*H2*: Secure attachment (SE) moderates the positive relationship between MW and well-being, such that entrepreneurs with a higher secure attachment to their leaders experience higher well-being (Mikulincer and Shaver, 2007; Pham et al., 2023; Warnock et al., 2024).

*H3*: Anxious attachment (AX) moderates the relationship between MW and well-being, with entrepreneurs with higher levels of AX experiencing a stronger positive effect of MW on well-being (Bodner et al., 2014; Jiang et al., 2019; Mikulincer and Shaver, 2007).

*H4*: Regional leaders significantly influence variation in entrepreneurial well-being (Jiang et al., 2019; Mikulincer and Shaver, 2007; Lisá and Greškovičová, 2023; Warnock et al., 2024).

## Methods

### Participants

The research involved entrepreneurs working as freelancers for a company specializing in financial products. The total number of entrepreneurs selling the company’s products was 500, all working as financial product salespeople. Of this group, 335 participants (67%) participated in the research. However, 35 participants were excluded due to incorrect responses to an attention-check question or because they were the sole representatives of their respective teams. Ultimately, data from 300 entrepreneurs, representing 60% of the company’s total sales force, were included in the analysis. Among these, 80% were female, 18% male, and 2% chose not to disclose their gender.

Participants were distributed across eight country regions, corresponding to 8 regional leaders. Within the participant group, 2% held middle management positions, 19% were sales managers, and 79% were salespeople. The average age of participants was 38.5 years (SD = 11.5), ranging from 21 to 72. The research included 50 teams, with an average of six employees per team and ranging from 2 to 12 members.

The company director invited the entrepreneurs to participate in the study. In return, the company received a report containing the average values of the studied variables for each sales team, along with a gift package of vitamin supplements for the sales teams. Participation in the study was voluntary, and all participants signed an online informed consent form.

Data collection was conducted online using the Qualtrics platform. The Ethical Committee of Faculty of Social and Economic Sciences, Comenius University Bratislava approved the research under protocol number 160–5/2023. Research is conducted in accordance with the Declaration of Helsinki (1964) and the ALLEA (2023). All data of individuals involved in the research are processed in accordance with Act No. 18/2018 Coll. on the Protection of Personal Data and Article 89(2) of the GDPR Directive. Participants and the company agreed to the use research data for a scientific publication.

### Measures

The Work and Meaning Inventory (WAMI), developed by Steger et al. (2012), is a 10-item scale to measure how individuals perceive their work as meaningful. It encompasses three dimensions: positive meaning, meaning-making through work, and a sense of contributing to the greater good. Sample items include statements such as “I have found a meaningful career” and “My work helps me better understand myself.” Responses are rated on a 5-point Likert scale, ranging from 1 (absolutely untrue) to 5 (absolutely true), with higher scores indicating greater perceived meaningfulness in work. In the current study, the WAMI demonstrated excellent internal consistency (*α* = 0.902).

The Workplace Attachment Questionnaire (WAQ), developed by Lisá and Greškovičová (2023), evaluates employee attachment at work through 13 items that focus on avoidant, anxious, and secure attachment styles with coworkers and supervisors. The WAQ is structured around three dimensions: (1) avoidant attachment to coworkers (AV), like “I avoid relationships with my coworkers,” (2) secure attachment to supervisors (SE), like “I have a good leader whom I trust” and (3) anxious attachment to both coworkers and supervisors (AX), like “I need to hear reassurance to keep me grounded at work.” Responses are provided on a 6-point Likert scale, from 1 (strongly disagree) to 6 (strongly agree). Reliability indices in the current study indicated high internal consistency (SE *α* = 0.985, AX *α* = 0.883, AV *α* = 0.875).

The Well-Being Index (WHO-5), validated by Topp et al. (2015), measures current mental well-being through five positively phrased items, such as “I have felt cheerful and in good spirits.” Responses are rated on a 6-point Likert scale (0 = at no time; 5 = all of the time), yielding a total raw score ranging from 0 to 25. This raw score is multiplied by 4 to generate a maximum score of 100. Higher scores reflect good well-being. In this study, the WHO-5 exhibited excellent reliability (*α* = 0.940).

### Data analysis

Statistical analyses began with calculating descriptive statistics, including means (M) and standard deviations (SD), to summarize the data distribution. Internal consistency and reliability of the scales were assessed using Cronbach’s alpha, with all variables demonstrating high reliability.

A bivariate Pearson correlation analysis was performed to identify relationships among variables. Subsequently, linear regression analysis tested the predictive power of the independent variable (MW, SE, AX, AV) on the dependent variable (Well-being), interactions (Wami × AV, Wami × SE, Wami × AX), and dedicated regional leaders. Regional leaders were treated as categorical fixed factors. Interactions were calculated using mean-centered values of the variables, a process achieved by subtracting the mean from each value. The approach mitigated multicollinearity and facilitated accurate interpretation of the interactions. Multicollinearity diagnostics (VIF values) were below 1.5, confirming no significant issues.

## Results

The means (M) and standard deviation (SD) for variables suggest a considerable spread across the variables, especially for WHO, which exhibited the highest variability (Table 1). Reliability analyses showed that all variables demonstrated high internal consistency, as indicated by their Cronbach’s alpha values. WHO had an alpha of 0.940, indicating excellent reliability. Wami and AV also exhibited high reliability with alphas of 0.902 and 0.875, respectively. SE showed the highest reliability with an alpha of 0.985, while AX had a slightly lower but acceptable alpha of 0.883.

**Table 1:** Correlation coefficients, means, standard deviations, and Cronbach’s alphas.

Variable	1	2	3	4	5
1. WHO	0.940				
2. Wami	0.397***	0.902			
3. AV	−0.187**	−0.351***	0.875		
4. SE	0.228***	0.308***	−0.234***	0.985	
5. AX	−0.082	−0.035	−0.023	0.146*	0.883
AM	69.587	40.611	7.398	26.041	16.426
SD	20.327	6.733	3.895	6.295	5.493

WHO, well-being; WAMI, meaningful work; AV, avoidant attachment to colleagues; SE, secure attachment to leader; AX, anxious attachment to leader/colleagues; AM, mean; SD, standard deviation. **p < 0.01; ***p < 0.001.

Bivariate Pearson correlations were computed to assess the relationships between the variables (Table 1). Correlation coefficients ranged from 0.023 to 0.397, indicating a low risk of common method bias. WHO positively correlated with Wami (*r* = 0.397, *p* < 0.001) and SE (*r* = 0.228, *p* < 0.001) but showed a small negative correlation with AV (*r* = −0.187, *p* < 0.01) and AX (*r* = −0.082, *p* > 0.05). Wami demonstrated a medium positive correlation with SE (*r* = 0.308, *p* < 0.001) and negative correlation with AV (*r* = −0.351, *p* < 0.001). No significant correlation was found between Wami and AX (*r* = −0.035, *p* > 0.05). AV was negatively correlated with SE (*r* = −0.234, *p* < 0.001). At the same time, AX showed a weak positive correlation with SE (*r* = 0.146, *p* < 0.05) and non-significant correlations with the remaining variables. WHO, Wami, and SE share significant positive relationships, whereas AV exhibits negative correlations with multiple variables.

In the linear regression model, we included the entrepreneurs’ leaders as categorical fixed factors, in addition to MW, attachment styles, and their interactions (Table 2). The model explained 26.8% of the variance in entrepreneurs’ well-being, as indicated by the *R*^2^ value. The adjusted *R*^2^ value was 0.223. The model was statistically significant *F*(14, 227) = 5.936, *p* < 0.001. MW was the strongest predictor of entrepreneurs’ well-being. It had a significant positive effect (*β* = 0.929; SE = 0.197; *t* = 4.726; *p* < 0.001), indicating that well-being improves as the perceived MW increases. SE to the leader also had a statistically significant positive effect (*β* = 0.417; SE = 0.205; *t* = 2.030; *p* = 0.044). The entrepreneurs who feel securely attached to their leaders experience better well-being. On the other hand, AV did not statistically significantly relate to well-being (*β* = −0.216; SE = 0.338; *t* = −0.638; *p* = 0.524), nor did AX, although it approached significance (*β* = −0.435; SE = 0.222; *t* = −1.957; *p* = 0.052).

**Table 2:** Regression coefficients.

Model		Unstandardized	Standard error	Standardized ^a^	*t*	*p*
H₀	(Intercept)	27.311	10.374		2.633	0.009
	Wami	0.966	0.198	0.313	4.882	< 0.001
	SE	0.433	0.204	0.132	2.124	0.035
	AV	−0.166	0.340	−0.031	−0.488	0.626
	AX	−0.400	0.225	−0.108	−1.780	0.076
	Inter1 (Wami × SE)	−0.027	0.027	−0.066	−0.989	0.324
	Inter2 (Wami × AV)	0.015	0.043	0.025	0.358	0.721
	Inter3 (Wami × AX)	0.079	0.033	0.148	2.408	0.017
H₁	(Intercept)	36.811	11.048		3.332	0.001
	Wami	0.929	0.197	0.301	4.726	< 0.001
	SE	0.417	0.205	0.127	2.030	0.044
	AV	−0.216	0.338	−0.040	−0.638	0.524
	AX	−0.435	0.222	−0.118	−1.957	0.052
	Inter1 (Wami × SE)	−0.032	0.027	−0.078	−1.171	0.243
	Inter2 (Wami × AV)	0.013	0.043	0.021	0.305	0.761
	Inter3 (Wami × AX)	0.086	0.033	0.161	2.626	0.009
	Leader (TT)	−13.254	6.047		−2.192	0.029
	Leader (TN)	−2.152	5.264		−0.409	0.683
	Leader (NR)	−10.965	4.613		−2.377	0.018
	Leader (ZA)	−8.776	5.328		−1.647	0.101
	Leader (BB)	3.058	5.222		0.586	0.559
	Leader (PO)	−8.743	4.585		−1.907	0.058
	Leader (KE)	−7.551	5.519		−1.368	0.173

a Standardized coefficients can only be computed for continuous predictors.

WHO, well-being; WAMI, meaningful work; AV, avoidant attachment to colleagues; SE, secure attachment to leader; AX, anxious attachment to leader/colleagues.

The interaction terms included in the model did not show significant effects, except for the interaction between MW and AX (*β* = 0.086; SE = 0.033; *t* = 2.626; *p* = 0.009). The interaction suggests that AX amplifies the effect of Wami on well-being. For entrepreneurs with higher levels of AX, the positive effect of MW on well-being is stronger. The relationship between Wami and well-being is not consistent across all levels of AX. When AX is low, Wami’s effect on well-being diminishes.

The slopes for Wami at different levels of AX (Low, Medium, and High) are statistically significant. At low AX, the relationship between Wami and well-being is weaker (slope = 1.83; SE = 0.41; *t* = 4.45; *p* < 0.001). The relationship is stronger at medium AX compared to low AX (slope = 2.26; SE = 0.58; *t* = 3.92; *p* < 0.001). Wami has a significant positive effect on well-being when AX is at an average level. At high AX, the relationship is the strongest, indicating that when AX is high, Wami has a much greater impact on the outcome (slope = 2.70; SE = 0.75; *t* = 3.60; *p* < 0.001). Wami has a significant positive effect on well-being when AX is high.

Several regional leaders demonstrated significant effects on well-being compared to the reference BA regional leader. Entrepreneurs under TT leader reported significantly lower well-being than the reference BA region (*β* = −13.254; SE = 6.047; *t* = −2.192; *p* = 0.029). Entrepreneurs under NR leader also had significantly lower well-being than the reference BA region (*β* = −10.965; SE = 4.613; *t* = −2.377; *p* = 0.018). Other regional leaders, such as TR, ZA, BB, PO, and KE, did not show statistically significant well-being differences compared to Bratislava.

According to the reviewers’ comments, objective team-level financial performance (sales index) was included in the regression model to enhance the validity of self-reported well-being outcomes. The extended model explained 26.1% of the variance in well-being. Entrepreneurs perceiving their work as meaningful report significantly higher well-being (*β* = 0.611; *p* = 0.011). Financially better-performing teams are associated with higher well-being among entrepreneurs (*β* = 0.273; *p* = 0.008). Entrepreneurs in the PO region reported significantly lower well-being than those in the reference region (*β* = −17.274; *p* = 0.006). Other attachment variables (SE: *β* = −0.079; *p* = 0.782; AV: *β* = −0.300; *p* = 0.460; AX: *β* = −0.371; *p* = 0.209), as well as their interactions with MW, were not statistically significant in the extended model. Once the objective financial performance indicator (sales index) was introduced, the previously significant AX × MW interaction became non-significant (*β* = 0.069; *p* = 0.123). The direct effect of SE declined in significance, indicating that economic factors can supersede SE to the leader in determining entrepreneurial well-being. Including objective performance may partially account for variance previously explained by psychological interactions.

To further control for demographic differences, gender was added to the extended regression model. The model remains statistically significant (*F*(16, 163) = 3.833; *p* < 0.001) and explains 27.3% of the variance in well-being, slightly improving upon the prior version with sales index only. In the extended regression model incorporating psychological variables, the team-level financial performance indicator (sales index), regional controls, and gender, three variables emerged as statistically significant predictors of entrepreneurs’ well-being: MW, sales index, and regional leader. MW had a significant positive effect on well-being (*β* = 0.587; *p* = 0.015), indicating that entrepreneurs who perceive their work as meaningful report higher levels of well-being. Similarly, objective team performance (sales index) was a significant predictor (*β* = 0.299; *p* = 0.005), suggesting that entrepreneurs working in financially better-performing teams experience greater well-being. Regarding regional leaders, entrepreneurs in the PO region reported significantly lower well-being than those in the reference region (*β* = −18.663; *p* = 0.003). Other variables in the model were not statistically significant: the attachment styles to the leader, SE, AV, and AX, as well as their interaction terms with MW. These effects did not reach significance when accounting for the stronger sales index and MW contributions. Likewise, gender (male vs. female) did not significantly predict well-being (*β* = 4.924; *p* = 0.217), suggesting no substantial gender differences in subjective well-being within the sample.

## Discussion

The results of the current study support Hypothesis 1 and confirm that MW is a robust predictor of entrepreneurial well-being. Entrepreneurs who perceive their work as a meaningful experience tend to experience better well-being, even when accounting for objective factors like team financial performance (measured by the sales index) and gender. This finding aligns with existing research that emphasizes the important role of MW in promoting psychological resilience, engagement, and overall life satisfaction (Allan et al., 2019; Steger and Dik, 2009; Martela and Pessi, 2018). Including objective performance in the regression model enhanced the ecological validity of findings by demonstrating that MW is not only a subjective construct but also relates to externally verifiable indicators of success. MW enhances well-being in high-demand environments, such as entrepreneurship (Allan et al., 2016; Steger and Dik, 2009).

### Secure attachment (hypothesis 2)

In the model focused solely on psychological variables, SE to leaders emerged as a significant predictor of well-being, supporting theoretical claims that SE fosters trust, emotional stability, and positive relational experiences (Mikulincer and Shaver, 2007; Pham et al., 2023). The findings reinforce prior research on the critical role of leadership in fostering a psychologically safe and MW environment (Grama and Todericiu, 2017; Rifa’i et al., 2024). However, the expected moderating role of SE on the MW and well-being relationship was not supported. A plausible explanation is that MW already encompasses many relational and motivational benefits associated with SE, limiting its additional moderating effect. When objective financial performance was introduced, SE lost its predictive significance. Financial success may be a more salient source of psychological security in structured sales environments than attachment to a leader. The diminished effect of SE also highlights potential conceptual overlap with MW, which reflects elements of perceived support, purpose, and efficacy.

### Anxious attachment (hypothesis 3)

The initial model, which included only psychological variables, also supported H3, indicating that AX moderates the relationship between MW and well-being, with MW having a larger positive effect on well-being for individuals with higher levels of AX. Prior research has suggested that individuals with anxious attachment styles may be more susceptible to cues of meaning and value in work and derive emotional regulation from meaningful engagement (Bodner et al., 2014; Jiang et al., 2019). However, the interaction lost its significance in the extended model, suggesting that objective performance measures may buffer or overshadow the psychological benefits of MW for anxiously attached individuals. That is, while MW may temporarily alleviate the insecurities of anxiously attached entrepreneurs, their well-being is also strongly linked to tangible indicators of success, such as sales performance. The finding highlights the importance of integrating subjective and objective dimensions when assessing well-being in entrepreneurial contexts.

AX’s amplifying effect may still be theoretically relevant. According to Karreman and Vingerhoets (2012), individuals with insecure attachment styles, including AX, often experience reduced well-being due to impaired emotion regulation. In a business context, where stable interpersonal support may be lacking, MW may serve as a surrogate regulatory mechanism, allowing individuals with AX to reinterpret stressors and create coherent, meaningful narratives about their work. Thus, MW’s intrinsic value and purpose may enhance well-being, particularly for AX entrepreneurs who actively seek validation and stability. As the significant moderator between MW and well-being, AX may be theoretically grounded in meaning-seeking as a compensatory coping strategy. Meaning-seeking models of coping emphasize cognitive reappraisal as a central mechanism for emotional adjustment in high-stress environments (Park and Folkman, 1997). Anxiously attached entrepreneurs may hyper-focus on work as a domain where they can assert value and receive implicit validation. Meaning-seeking behaviors enhance emotional regulation and reduce anxiety, contributing to well-being (Pfund et al., 2024). Furthermore, meaning-making has been shown to serve as a core mechanism of posttraumatic growth in populations facing profound psychological stress, such as combat veterans, by transforming distress into adaptive narratives (Larner and Blow, 2011). Similarly, MW may allow AX entrepreneurs to reframe their professional challenges as purposeful, thereby buffering uncertainty and enhancing psychological resilience. AX individuals are susceptible to positive and negative relational cues (Mikulincer and Shaver, 2007). Their heightened emotional reactivity makes them particularly vulnerable to perceived rejection or unpredictability, and they are responsive to support, validation, and signals of meaning. In this light, MW may function as a positive psychological cue that symbolizes value, coherence, and purpose, temporarily alleviating the underlying anxiety associated with attachment insecurity.

### Regional leadership (hypothesis 4)

The study provides partial support for H4. In both the basic and extended models, some regional leaders had significantly lower well-being scores than the reference leader. Regional leadership continues to exert an independent effect, even after controlling for performance (as measured by the sales index) and demographic variables.

A closer look at the assessment center results for the company suggests that differences in leadership style and regional context may help explain the well-being prediction in some regions. In one of the significant regions (not explicitly mentioned due to anonymity), the assessment center indicated a more directive or controlling leadership style, with limited room for team members’ autonomy and creativity. Such an environment can reduce employees’ sense of ownership and psychological comfort, thus contributing to lower well-being. In contrast, in another region, where the leader received very positive ratings, employees enjoy more significant support, encouragement, and open communication, consistent with the region’s higher well-being ratings. Meanwhile, the other region, led by a leader who advocates for rapid innovation and improvement, presents another possible mismatch. While the leader’s focus on creativity and forward-thinking initiatives may be beneficial in some contexts, employees in a traditional insurance environment may not be accustomed to constant innovation or self-directed problem-solving. This cultural or motivational mismatch can lead to uncertainty or stress, which in turn can affect the overall well-being of employees. In addition to leadership style, region-specific market factors such as local competition, sales pressure, or historical performance expectations can also contribute to increased stress levels among team members. Qualitative interviews or focus groups with regional employees and leaders would clarify precisely how these factors interact. Such discussions could shed light on whether leadership style creates friction, whether innovation demands are perceived as overwhelming or insufficiently supported, or whether regional market conditions (such as sales targets and competition) contribute to lower well-being. Future studies should also consider including direct measures of leadership behaviors and perceived psychological safety to isolate the effects of leadership better.

What leadership practices simultaneously support MW and employee well-being? Research suggests these practices are deeply rooted in psychological safety. Inclusive leadership enhances MW by fostering open communication and inclusivity, enabling employees to feel valued and secure, thereby increasing their perception of MW (Rifa’i et al., 2024). Psychological safety further strengthens the positive relationship between inclusive leadership and MW. Ethical leadership also positively influences MW by promoting transparency and fairness. Effective communication mediates the relationship between ethical leadership and MW, emphasizing the importance of transparent and honest interactions in fostering an environment that supports MW (Mosquera et al., 2024).

The current study extends attachment theory to entrepreneurship within structured organizational systems, offering new insights into how attachment dynamics unfold in hybrid work contexts where autonomy and hierarchy coexist. Attachment theory has been widely applied in employee-focused research (Lisá and Greškovičová, 2023), where attachment to the leader is related to citizenship organizational behavior, or leader-rated team performance. Entrepreneurs in structured sales networks are technically independent actors but are also embedded in a corporate hierarchy that includes regional leaders and performance supervision. This dual status creates a different psychological environment: the need for security and relational validation (key to attachment processes) must be balanced with expectations of autonomy, initiative, and entrepreneurial autonomy. The results of the current study suggest that SE to leaders may also enhance well-being in an entrepreneurial context but that its predictive power is reduced when objective financial performance (sales index) is taken into account, in contrast to traditional work environments where SE has been shown to predict engagement and citizenship organizational behavior reliably (Lisá et al., 2021).

### Limitations and future research implications

The study was conducted within a single industry, financial services, which presents specific contextual factors that may have influenced the results. In structured sales networks (Arndt and Harkins, 2013), entrepreneurial activity is characterized by hierarchical oversight, performance-based incentives, and high sales pressure. These characteristics underscore the significance of secure managerial relationships and interpersonal dynamics in shaping perceptions of MW and overall well-being. Caution is therefore warranted in generalizing findings to other entrepreneurial settings, such as technology startups, creative industries, or fully autonomous freelance models, where leadership structures, performance metrics, and cultural norms differ significantly. Future research should examine whether similar patterns of interactions between MW, attachment styles, and well-being emerge in less hierarchical or more innovation-driven sectors. It is important to note that the results may not generalize even to employees within the same financial sector who receive a stable, fixed salary regardless of individual or team performance.

A significant gender imbalance in the sample may influence the interpretation of results, particularly concerning attachment styles and the perception of MW. The gender distribution (80% female, 18% male, 2% undisclosed) is a critical factor that may impact the study’s findings and their broader implications. Women are more likely to report higher levels of AX and emphasize the relational and purpose-driven aspects of work, which affects their experience of MW (Bodner et al., 2014; Herr et al., 2023). Gender-specific attachment dynamics suggest that women tend to derive more meaning from relational support and collaborative environments than men (Wallace, 2019; Jiang et al., 2019). Female participants may resonate more strongly with MW dimensions, such as “creating meaning through work” and “contributing to a greater good,” which could potentially skew MW scores compared to a gender-balanced sample.

Future interventions should evaluate whether methods for enhancing MW, such as cultivating a sense of purpose and connection, are equally effective for male participants, who may prioritize different work values, including financial rewards (Burbano et al., 2023). Gender differences in attachment styles further complicate interpretation; women are more likely to report higher AX levels, while men report higher AV (Euler, 2020). The predominantly female sample may explain the pronounced moderating role of AX observed in the current study. Women’s heightened sensitivity to emotional and relational cues may further magnify the psychological impact of MW. In contrast, men’s potentially greater focus on financial success or avoidance strategies may alter their perceptions of MW. These potential gender-specific mechanisms highlight the importance of replicating findings in more balanced or male-dominated samples. Support strategies targeting entrepreneurs with AX (reassurance and positive feedback) may disproportionately cater to female participants, potentially neglecting the avoidant tendencies more common among men (Euler, 2020). To address this gap, interventions for men should focus on building SE, fostering trust, and reducing discomfort with closeness (the key elements of avoidant attachment).

Women’s heightened sensitivity to social aspects of well-being might amplify the significance of leader-entrepreneur relationships in predicting overall well-being (Topp et al., 2015). Utilizing tools such as WHO-5, which emphasize positive emotions and relational dynamics, may favor female perspectives on well-being. Future studies should evaluate whether existing well-being measures adequately capture dimensions more salient for male entrepreneurs, such as financial stability (Euler, 2020; Romney et al., 2024), and replicate the current study’s results in more gender-balanced samples.

While the study effectively explores the interplay between MW and workplace attachment styles, it does not comprehensively address other critical moderating factors, such as organizational culture and psychological safety. An inclusive, collaborative culture aligned with shared values can amplify the positive effects of MW, while a toxic or misaligned culture could exacerbate the adverse impacts of insecure attachment styles (Kahn, 2007). Psychological safety, characterized by the freedom to express oneself without fear of negative consequences (Edmondson, 1999), is another crucial variable that may mediate/moderate the relationship between attachment styles and MW. Entrepreneurs with SE are likely to experience higher psychological safety, strengthen MW, and improve well-being. The absence of psychological safety could diminish the link between MW and well-being, particularly for individuals with AX or AV styles (Rifa’i et al., 2024; Edmondson, 1999).

The cross-sectional design severely limits the ability to draw causal inferences. While MW has been shown to predict well-being (Allan et al., 2019; Lips-Wiersma et al., 2023), it is equally plausible that individuals with higher baseline well-being are more likely to perceive their work as meaningful. The potential for reverse causality should be explicitly acknowledged. For example, entrepreneurs who experience better life and work satisfaction (Duarte-Lores et al., 2023; Hu and Hirsh, 2017) or optimism (Bunjak et al., 2022) are more likely to interpret their work through a meaningful lens.

Additionally, the study does not control for the influence of third variables, such as stable personality traits (Barrick et al., 2013), which are known to shape well-being and perceptions of meaning in work. These unmeasured variables (such as tenure) could confound the observed relationships and should be considered in future research designs. For example, the sales index emerged as a significant positive predictor of well-being, suggesting that entrepreneurs working in higher-performing teams report higher psychological well-being. The finding underscores the importance of triangulating subjective measures such as MW with objective performance indicators. Financial success may serve as a validating feedback mechanism that reinforces the perceived meaningfulness and legitimacy of entrepreneurial efforts. Future researchers can employ multilevel or longitudinal designs to disentangle temporal and contextual influences.

Tracking changes in MW, attachment security, and well-being over time would enable stronger causal inferences and the identification of developmental trajectories. In addition, experimental or quasi-experimental designs, such as interventions aimed at enhancing MW (for example, through job crafting, leadership training, and meaning-centered coaching), could help establish causal relationships and test whether changes in MW precede improvements in well-being. Integrating such approaches would strengthen the robustness and practical implications of the findings.

### Practical implications

Targeted training could focus on building trustworthy relationships through support, empathy, and transparent communication (London and Zobrist, 2024) to enhance SE skills among leaders. These factors bolster entrepreneurs’ stress management capabilities and contribute significantly to their well-being. Reitz et al. (2017) suggested that implementing mentorship programs would enable experienced leaders to guide less seasoned entrepreneurs, helping them derive greater meaning from their work and improving their well-being.

Organizations can foster mentorship by creating opportunities and peer-support systems for regional leaders. Pairing high-performing leaders from regions with those from regions with lower well-being scores could be particularly effective. Furthermore, the results highlight that leaders in certain regions have a significant impact on entrepreneurs’ well-being. Companies should implement robust evaluation mechanisms to monitor leadership styles and their effects on team well-being. Training regional leaders to promote psychological safety, inclusivity, and fairness is essential, as these elements enhance the sense of MW (Rifa’i et al., 2024; Prihartati et al., 2023). Entrepreneurs with high levels of AX could also benefit from tailored feedback designed to reduce anxiety (Gregersen, 2023). Access to coaching or psychological counseling services may help them manage their need for constant reassurance. Moreover, providing regular feedback and clearly defined goals can alleviate anxiety while keeping their focus on meaningful aspects of work (Wirzberger et al., 2024).

Empirical research underscores the importance of MW for entrepreneurs. Organizations can enhance employee well-being by creating environments that offer autonomy, align work with personal values, and ensure that employees perceive their work as contributing to a larger purpose (Lips-Wiersma et al., 2023). For instance, organizations and schools should prioritize improving job characteristics to enhance the psychological meaningfulness of workers and educators, as well as their job attachment. These could include granting greater autonomy, offering constructive feedback, and fostering a supportive work environment (Akmadelita and Kusumaputri, 2018).

Organizations should adopt leadership styles that balance well-being and performance (Hart, 2020). By fostering a healthier organizational culture, companies can integrate well-being metrics into performance evaluations for regional leaders. They could involve tracking correlations between leadership behaviors and regional well-being indicators, or rewarding leaders who demonstrate strong team support, as evidenced by improvements in team well-being and MW scores. Standardizing communication practices is another viable strategy. It would ensure consistency across regions through protocols emphasizing clear, positive, and meaningful feedback. Such uniformity can help reduce regional disparities and promote a cohesive culture of well-being.

Leadership training should focus on enhancing secure attachment through trust and empathy and aligning leadership practices with performance goals. Leaders must cultivate psychological safety and clarity of goals, as these elements significantly impact overall well-being. For anxiously attached entrepreneurs, interventions such as regular positive feedback, meaningful coaching, and supportive communication can enhance MW but should be complemented by performance support tools to sustain well-being. Organizations should integrate performance-based metrics into well-being dashboards, recognizing that financial success is not only an outcome but also a contributor to mental health in entrepreneurial settings.

## Conclusion

MW is a stable and meaningful predictor of well-being, regardless of workplace attachment style or gender. However, the relationship to workplace attachment styles, mainly SE and AX, may be conditional rather than universal, influenced by contextual factors such as financial performance or leadership quality. Psychological variables interact with structural conditions to shape well-being. The loss of significance for AX and SE when objective performance is included may indicate that entrepreneurial well-being is not purely a function of individual differences or internal motivation but is equally shaped by economic realities and team context.

## Data Availability

The raw data supporting the conclusions of this article will be made available by the authors, without undue reservation.
